# Phenotypically Adapted Mycobacterium tuberculosis Populations from Sputum Are Tolerant to First-Line Drugs

**DOI:** 10.1128/AAC.01380-15

**Published:** 2016-03-25

**Authors:** Obolbek Turapov, Benjamin D. O'Connor, Asel A. Sarybaeva, Caroline Williams, Hemu Patel, Abdullaat S. Kadyrov, Akpay S. Sarybaev, Gerrit Woltmann, Michael R. Barer, Galina V. Mukamolova

**Affiliations:** a Department of Infection, Immunity and Inflammation, University of Leicester, Leicester, United Kingdom; b Empath Pathology Services, Department of Clinical Microbiology, University Hospitals of Leicester NHS Trust, Leicester, United Kingdom; c National Center of Phthisiology, Akhunbaeva, Bishkek, Kyrgyzstan; d Kyrgyz Indian Mountain Biomedical Research Center, Togolok Moldo, Bishkek, Kyrgyzstan; e Department of Respiratory Medicine, Glenfield Hospital, Leicester, United Kingdom

## Abstract

Tuberculous sputum contains multiple Mycobacterium tuberculosis populations with different requirements for isolation *in vitro*. These include cells that form colonies on solid media (plateable M. tuberculosis), cells requiring standard liquid medium for growth (nonplateable M. tuberculosis), and cells requiring supplementation of liquid medium with culture supernatant (SN) for growth (SN-dependent M. tuberculosis). Here, we describe protocols for the cryopreservation and direct assessment of antimicrobial tolerance of these M. tuberculosis populations within sputum. Our results show that first-line drugs achieved only modest bactericidal effects on all three populations over 7 days (1 to 2.5 log_10_ reductions), and SN-dependent M. tuberculosis was more tolerant to streptomycin and isoniazid than the plateable and nonplateable M. tuberculosis strains. Susceptibility of plateable M. tuberculosis to bactericidal drugs was significantly increased after passage *in vitro*; thus, tolerance observed in the sputum samples from the population groups was likely associated with mycobacterial adaptation to the host environment at some time prior to expectoration. Our findings support the use of a simple *ex vivo* system for testing drug efficacies against mycobacteria that have phenotypically adapted during tuberculosis infection.

## INTRODUCTION

Tuberculosis (TB) remains a major infectious disease that claims nearly 2 million lives annually (1). Both the requirement for prolonged treatment with a combination of drugs and the rise of multiple and extensively drug-resistant strains emphasize the need for effective new drugs. Mycobacterium tuberculosis adopts multiple physiological states during infection, and this provides part of the rationale for use of multiple drugs (2).

Sputum samples from patients are widely used for the microbiological diagnosis of TB by microscopy, culture, and molecular techniques (3); they also represent a valuable source of mycobacteria phenotypically adapted to the *in vivo* environment. Sputum mycobacteria display a “fat and lazy persister-like” phenotype with reduced metabolic activity, prominent lipid bodies, and a unique transcriptomic signature suggesting adaptation to slow growth or nongrowth (4). Application of growth assays allowed us to establish that mycobacterial populations in sputum produce distinct patterns of growth referred to here as “plateable” (CFU producing), “nonplateable” (liquid culture dependent) (5), and “Rpf dependent” (growth dependent on liquid medium supplemented with recombinant resuscitation-promoting factor [Rpf] or Rpf-containing culture supernatant [SN]) (6).

These distinct growth phenotypes may reflect the different *in vivo* populations that must be eliminated by chemotherapy and may also underpin well-known phenomena, such as early bactericidal activity and relapse after treatment. Specific aspects of the *in vivo* environment are likely to be the major factors driving phenotypic heterogeneity (7, 8). Therefore, it may be critical to assess activities against *in vivo*-adapted bacilli to understand and predict the different responses of M. tuberculosis subpopulations to drug treatment. Mouse infection models have been used extensively to assess anti-TB drugs; however, they do not precisely represent the human *in vivo* environment and have certain well-recognized limitations (9).

We propose that direct study of M. tuberculosis cells in sputum provides an important opportunity to demonstrate aspects of the chemotherapeutic efficacy of drugs and thereby facilitate drug development. The main challenge in direct assessment of M. tuberculosis in sputum samples for the response to drugs is posed by the difficulty in reliable preservation of heterogeneous M. tuberculosis populations for multiple assays. While freezing and storage of *in vitro*-grown M. tuberculosis cultures at −70 to −80°C did not influence mycobacterial viability in several studies (10–12), relatively little is known about viability of M. tuberculosis in sputum samples subjected to freezing. Tessema et al. showed that storage of smear-positive sputum samples at −20°C for up to 180 days sustained an overall culture positivity rate of 90% on multiple media (13), but those authors did not compare bacterial numbers before and after storage. Other sputum preservation methods, such as addition of 1% cetylpyridinium chloride, also have certain limitations and influence acid-fast staining properties (14).

In this proof-of-concept study, we have developed a simple procedure for cryopreservation of heterogeneous M. tuberculosis populations from sputum samples and applied this to establish a novel direct drug tolerance assay using *ex vivo* mycobacteria from sputum.

## MATERIALS AND METHODS

### Patients.

The study was approved by the Leicestershire, Northamptonshire, and Rutland Research Ethics Committee (07/Q2501/58). Consenting patients who produced smear-positive sputum provided samples for the study before onset of chemotherapy. The presence of M. tuberculosis in all samples was confirmed by microscopy and culture. The identity of M. tuberculosis strains and their drug susceptibility profiles were determined as described below. Altogether, samples from 15 patients were used in this study. Samples were collected in Leicester (United Kingdom) and Bishkek (Kyrgyzstan). Sputum samples were kept at 4°C for up to 7 days before decontamination and freezing, as previously described (6).

### Bacterial strains.

M. tuberculosis H37Rv was grown in 7H9 medium supplemented with 10% (vol/vol) oleic acid-albumin-dextrose-catalase (Becton, Dickinson and Company), 0.05% (wt/vol) Tween 80, and 0.2% (vol/vol) glycerol for production of culture supernatant and for antimicrobial tolerance assays. This medium is referred to as supplemented 7H9 medium. M. tuberculosis isolates from sputum were grown on 7H10 agar or in 7H9 supplemented medium. Bacteria were grown in Sterilin polypropylene 30-ml universal containers with 5 ml of medium, Falcon 50-ml conical centrifuge tubes with 10 ml of medium, or in 2-liter Nunc roller bottles with 200 ml of medium. Tubes were incubated at 37°C with shaking (100 rpm). Mycobacterial strains were typed using mycobacterial interspersed repetitive-unit–variable-number tandem-repeat (MIRU-VNTR) analysis (15) as described previously (6).

### Preparation of culture supernatant.

M. tuberculosis H37Rv was cultured in supplemented 7H9 medium to an optical density at 580 nm (OD_580_) of 0.6 to 0.8. Cultures were centrifuged at 3,000 × *g* for 20 min, and supernatant was double filtered using a 0.20-μm VWR filtration system. Aliquots of 10 or 25 ml were freeze-dried using an AdVantage Plus ES-53 freeze-drier (Biopharma Process Systems Ltd., United Kingdom). Freeze-dried supernatant preparations were kept at −80°C for up to 6 months. As shown in Fig. S1 in the supplemental material, freeze-drying preserved resuscitation activity. For experiments, freeze-dried supernatant was reconstituted in sterile deionized water and kept on ice for 30 min to allow the dried material to completely dissolve. The dissolved supernatant was kept on ice and used within 1 h.

### Decontamination and storage of sputum samples.

Sputum samples were decontaminated as described previously (6). Briefly, 1 volume of sputum was mixed with 1 volume of 4% (wt/vol) NaOH and incubated at room temperature for 15 min. The mixture was neutralized with 2 volumes of 14% (wt/vol) KH_2_PO_4_. Decontaminated samples were centrifuged at 4,000 × *g* for 20 min and resuspended in the original volume of 10% (vol/vol) sterile glycerol, phosphate-buffered saline (PBS), or 200 mM trehalose. The mixture was passed through a 23-gauge blunt needle (Harvard Apparatus, United Kingdom) 5 times to maximize homogeneity and disperse potential mycobacterial aggregates (16, 17). The homogenized decontaminated M. tuberculosis cells from sputum samples were frozen and stored at −80°C in 0.5-ml aliquots. Sputum is often a viscous and clumpy material, and this may create an unequal distribution of mycobacteria and lead to high variability between counts. To address this, we introduced an additional step for the homogenization of sputum by passing the treated sample through blunt fine-gauge needles. Application of this method ensured minimal variation of bacterial counts between frozen aliquots of sputum samples (see Fig. S2 in the supplemental material).

### Growth assays.

Previous studies revealed three M. tuberculosis populations with different conditions for cultivation: (i) “plateable” populations grow on solid agar and numbers of plateable M. tuberculosis are determined by counting CFU; (ii) “nonplateable” populations grow in liquid medium and their numbers are assessed by most-probable-number assays in liquid 7H9 medium (MPN); (iii) “supernatant-dependent” M. tuberculosis cells can only grow in liquid medium supplemented with Rpf-containing SN and they are enumerated by MPN assay in 7H9 liquid medium supplemented with Rpf-containing culture supernatant (MPN_SN) (6). For CFU counts, mycobacteria were serially diluted in supplemented 7H9 medium and plated on 7H10 agar (Becton, Dickinson and Company). MPN counts were assessed in supplemented 7H9 medium, and MPN_SN counts were done in 7H9 supplemented with 50% (vol/vol) culture supernatant. The BBL MGIT PANTA antibiotic mixture (Becton, Dickinson and Company) was added to all media used for growth assays as recommended by the manufacturer. Agar and MPN plates were incubated at 37°C for up to 12 weeks. MPN counts were calculated as described previously (6).

### Drug tolerance assays.

Tolerance for each drug was assessed using four sputum samples from four separate patients. These samples were designated S6, S19, S20, and S21. Each sample was decontaminated, homogenized, and frozen in aliquots as described above. Decontaminated stored sputum samples (0.5-ml aliquots) were defrosted at room temperature and centrifuged for 20 min at 4,000 × *g*. The supernatant was discarded to minimize possible effects of the storage medium on M. tuberculosis responses to drug treatment, and each pellet was resuspended in 0.5 ml of supplemented 7H9 medium. Falcon tubes containing 10 ml of supplemented 7H9 medium with PANTA and an appropriate drug were inoculated with 0.5 ml of resuspended pellet. Antimicrobials were added to achieve the following final concentrations (in micrograms per milliliter): streptomycin, 10 and 20; rifampin, 1 and 5; ethambutol, 10 and 20; isoniazid, 1 and 10; pyrazinamide, 40 and 100. Pyrazinamide was tested at pH 5.6 and 6.8; appropriate controls were included for both conditions. Tubes were sealed and incubated at 37°C with shaking (100 rpm). M. tuberculosis CFU, MPN, and MPN_SN counts were determined at time zero (sample without drugs only) and after 3 and 7 days of incubation. At 3 days, half of the incubated sample (5 ml) was transferred into a separate Falcon tube and centrifuged at 4,000 × *g* for 20 min. Supernatants were discarded and pellets were resuspended in 0.5 ml of 7H9 medium; the resuspended pellets were used for determination of viable counts. Experiments were done in duplicate. Drug-free controls in media with standard pH 6.8 and the lower pH of 5.6 were included. While M. tuberculosis did not show significant replication in the low-pH medium, under standard conditions we detected an increase in CFU counts after 7 days of incubation. Therefore, drug effects are shown as the percent survival at the times indicated with respect to the counts determined in the inoculum. Survival percentages (%SR) were calculated separately for days 3 and 7 using the following formula: %SR = (viable count with treatment/viable count at zero time point) × 100%. The assay abbreviations, their target populations, and their interpretation are summarized in Table 1.

**TABLE 1:** Assay designs and interpretation

Assay	M. tuberculosis population targeted^*a*^	Basis of population size estimate	Interpretation
CFU	Plateable	CFU count	Population capable of forming colonies
MPN^*b*^	Nonplateable	MPN count	Population that grows in liquid medium
MPN_SN	SN dependent (putatively Rpf dependent)	MPN_SN count	Population dependent on Rpf-containing culture supernatant to grow

a For each target population, the proportion (percent) surviving at the end of drug exposure was estimated as follows: 100% × (count at day 3 or 7/count at time zero). The assays detect overlapping populations, but the degree of overlap is unknown.

b The MPN is usually higher than the CFU count in studies based on *in vitro*-grown bacteria; however, the MPN is often lower in sputum, and we have previously attributed this to an inhibitory activity (6).

Control experiments were conducted to compare antimicrobial tolerance of M. tuberculosis from sputum with those of the H37Rv laboratory strain and *in vitro*-passaged sputum isolates. H37Rv was grown in supplemented 7H9 medium to an OD_580_ of 0.8 and used for drug treatments as described above. Sputum isolates were passaged twice in supplemented 7H9 medium prior to drug tolerance assays. To assess the effects of the decontamination procedure and freezing on M. tuberculosis cultures, H37Rv and the S20 sputum isolate grown in 7H9 medium to an OD_580_ of 0.8 were subjected to the decontamination procedure and frozen in 10% glycerol as described above. In control experiments, grown M. tuberculosis cultures were spiked in nontuberculosis sputum followed by decontamination and freezing. In all experiments, M. tuberculosis cells were inoculated at ∼5 × 10^5^ to 1 × 10^6^ CFU/ml.

### Strain typing and indirect drug susceptibility testing.

Strains were typed using MIRU-VNTR, and their drug susceptibility was tested using an indirect method described previously (15, 18) Briefly, mycobacterial strains were plated on 7H10 agar containing rifampin (1.0 μg/μl), isoniazid (0.2 μg/μl), ethambutol (5.0 μg/μl), or streptomycin (2.0 μg/μl), and a drug-free control was included. Plates were incubated at 37°C and checked weekly, and at 3 weeks the number of colonies in the drug-containing plates was compared to the number of colonies in the drug-free plates. No colonies were detected on plates containing drugs.

## RESULTS

### Influence of storage medium on cryopreservation of M. tuberculosis populations from sputum.

**FIG 1:**
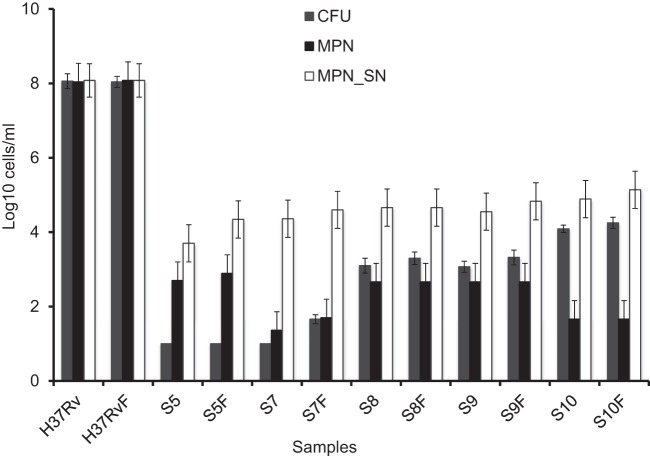
Comparison of counts for mycobacterial populations obtained from sputum before and after freezing in glycerol. Samples were decontaminated and frozen in 10% glycerol. M. tuberculosis counts were assessed before (H37Rv, S5, S7, S8, S9, and S10) and after (H37RvF, S5F, S7F, S8F, S9F, and S10F) freezing and defrosting. Error bars indicate 95% limits of confidence for MPN counts and standard deviation values for CFU counts. Note that because the MPN counts in all samples apart from S5 were the same or less than the CFU counts, no nonplateable M. tuberculosis cells were detected in the baseline samples, while a significant excess of SN-dependent bacilli was present in each sample.

Our initial experiments indicated that freezing of nondecontaminated samples produced highly variable results between sputum aliquots (data not shown). Therefore, we explored conditions for optimal preservation and storage of decontaminated homogenized sputum samples. Table S1 in the supplemental material summarizes data on survival of sputum M. tuberculosis under three different solutions: (i) PBS; (ii) 200 mM trehalose in water; (iii) 10% (vol/vol) glycerol in water. Our data showed that storage of decontaminated samples in all media resulted in significant preservation of colony-forming and SN-dependent mycobacteria. In most cases, freezing was associated with an increase in viable counts (both on agar and in liquid), and the highest counts were achieved in 10% glycerol. These observations suggested that freezing and subsequent centrifugation (see Materials and Methods) may remove the inhibitory activity previously found in sputum in some cases (6). Additional experiments with 5 separate sputum samples demonstrated that the glycerol solution is a satisfactory storage medium for the preservation of M. tuberculosis populations in sputum. Figure 1 demonstrates that viable counts of mycobacteria recovered from sputum samples frozen in glycerol were comparable to or higher than those from samples processed before freezing and that the relative amounts detected by the three culture methods were preserved. The ability to cryopreserve M. tuberculosis populations in sputum marks an important milestone which enabled us to study drug tolerance patterns in M. tuberculosis populations.

### Bactericidal activity of first-line drugs on M. tuberculosis populations in sputum.

**FIG 2:**
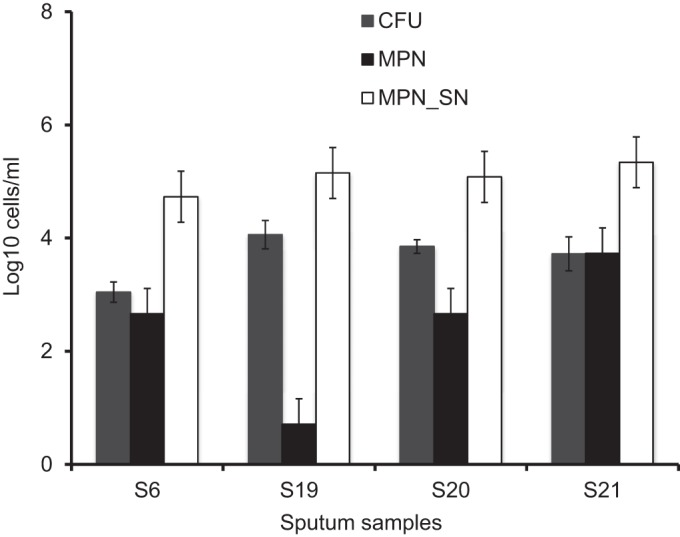
Characterization of mycobacterial populations in sputum from four samples selected for antimicrobial treatment experiments. Samples were decontaminated and stored as described in Materials and Methods. Error bars indicate 95% limits of confidence for MPN counts.

**FIG 3:**
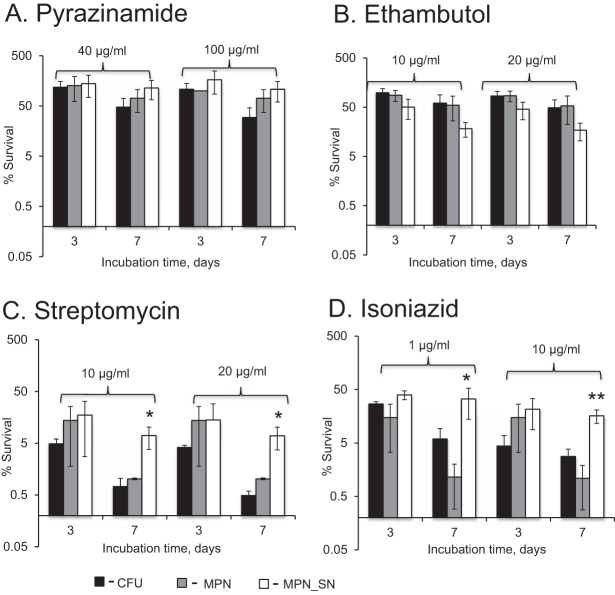
Investigation of drug effects on the viability of mycobacterial populations isolated from sputum. (A) Pyrazinamide; (B) ethambutol; (C) streptomycin; (D) isoniazid. The percent survival was calculated using the following formula: [(viable count_after exposure_)/(viable count_before exposure_)] ×100%. Mean values for 4 sputum samples (S6, S19, S20, and S21) are shown; error bars indicate standard deviations. *, MPN_SN survival values were statistically significantly different from CFU and MPN survival values (*P* < 0.05, one-way ANOVA); **, MPN_SN survival values were statistically significantly different from CFU and MPN survival values (*P* < 0.01, one-way ANOVA).

**FIG 4:**
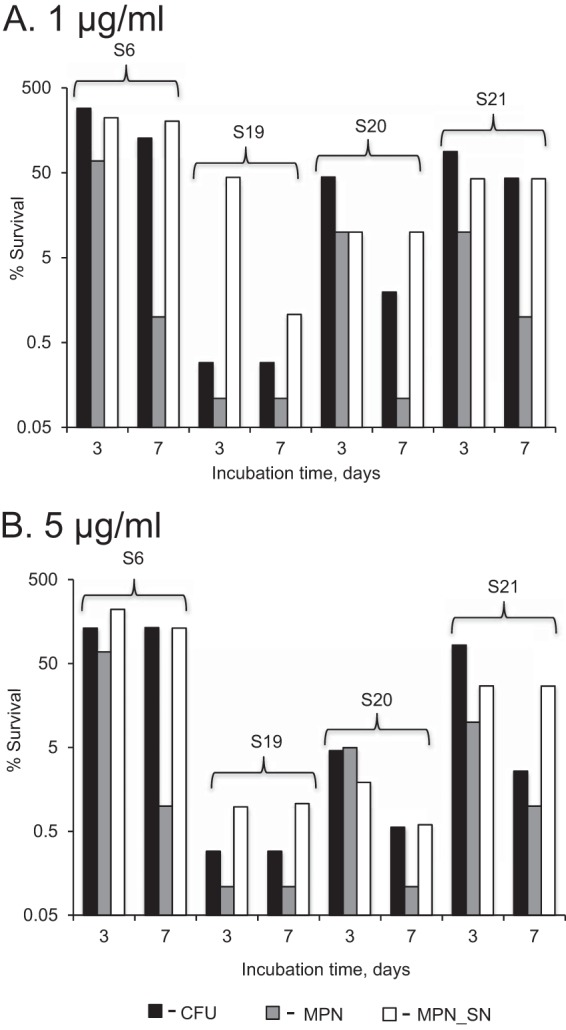
Rifampin has a variable effect on M. tuberculosis obtained from sputum. Mycobacteria were treated with 1 μg/ml (A) or 5 μg/ml (B) rifampin for 3 or 7 days. CFU, plateable M. tuberculosis; MPN, M. tuberculosis grown in liquid; MPN_SN, M. tuberculosis grown in the presence of culture supernatant.

To investigate the effect of drugs on various M. tuberculosis subpopulations, we selected high-volume sputum samples from 4 patients. As shown in Fig. 2, SN-dependent populations dominated all four sputum samples. Sensitivity of the isolates from these samples to growth inhibition by all first-line agents was confirmed by standard susceptibility testing (18) (see Materials and Methods). Antimicrobial tolerance of the M. tuberculosis populations was assessed separately for each sputum sample, and the results are summarized in Fig. 3 and 4.

Consistent patterns of efficacy were observed in all tested sputa with respect to all agents except rifampin. Therefore, the results of the assays based on inocula from four sputa are displayed (as the mean ± standard deviation [SD] percent survival) (Fig. 3), while those for rifampin are displayed separately (Fig. 4). Pyrazinamide showed no effect at 3 days and a modest bactericidal action at 7 days (<1 log_10_ reduction) (Fig. 3A). In contrast, streptomycin showed a clear effect on all M. tuberculosis populations at 7 days, with ∼2.5 log_10_ reductions in the CFU and MPN assays and ∼1 log_10_ reduction in the MPN_SN assay (Fig. 3C). Importantly, the MPN_SN assay revealed a significantly greater tolerance to streptomycin than did CFU-producing and liquid-grown M. tuberculosis (*P* < 0.05, one-way analysis of variance [ANOVA]). Like pyrazinamide, ethambutol only displayed modest bactericidal effects (<1 log_10_ reduction), which was most prominent at 7 days but with some evidence of greater efficacy revealed in the MPN_SN assay (Fig. 3B); however, this was not significant (*P* > 0.05, one-way ANOVA). Isoniazid showed dose-dependent bactericidal effects (1 to 2 log_10_ reductions) that were clearly greater in the CFU than in the MPN_SN assay (Fig. 4C and D). Interestingly, MPN counts were substantially reduced after 7 days of treatment with both concentrations. Similar to the results with streptomycin, the MPN_SN assay showed greater tolerance to both concentrations after 7 days than did the CFU or MPN assays. Rifampin produced highly variable results, ranging from complete tolerance (sample S6) to significant bactericidal activity (sample S19) (Fig. 4A and B). Although the responses were variable, we noted three particular features: (i) the MPN-assayed M. tuberculosis isolates were least tolerant to rifampin in all samples tested, supporting previously published data by Bowness and colleagues showing higher killing of M. tuberculosis by rifampin in liquid culture (19). (ii) In all but one sample exposed to 1 μg/ml (S19) (Fig. 4A), the MPN_SN assay showed no additional decline in survival at day 7 compared to day 3. (iii) Dose-dependent effects (greater reductions at higher doses) were apparent in the MPN_SN assay in samples S19 and S20 (Fig. 4A and B).

### *In vitro*-grown H37Rv M. tuberculosis and passaged sputum isolates are less tolerant by CFU assay compared to M. tuberculosis in sputum.

**FIG 5:**
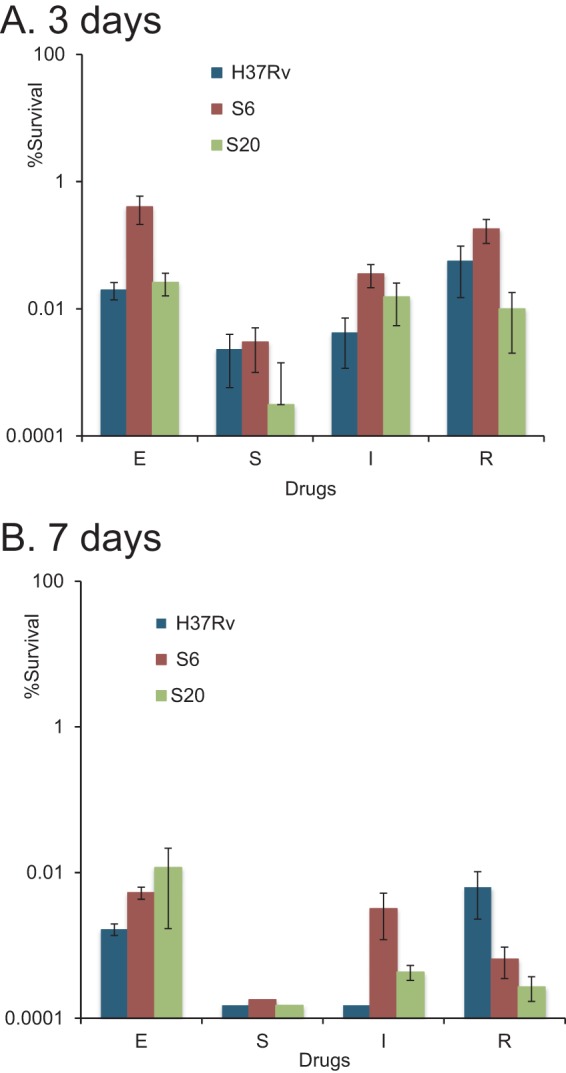
Bactericidal effects of drugs on plateable M. tuberculosis H37Rv and *in vitro*-grown sputum isolates. Log-phase bacteria were inoculated in 7H9 supplemented medium and treated with drugs for 3 (A) and 7 (B) days. S, streptomycin, 20 μg/ml; E, ethambutol, 20 μg/ml; I, isoniazid, 10 μg/ml; R, rifampin, 5 μg/ml. The limit of detection was 0.00015%.

The experiments described above demonstrated that substantial populations of plateable M. tuberculosis in sputum could tolerate bactericidal drugs. Further control experiments were conducted to clarify whether this property was attributable to phenotypic or genotypic differences. We first compared the responses of M. tuberculosis H37Rv and isolates from two of our sputum samples passaged twice in liquid medium for their responses to streptomycin, ethambutol, isoniazid, and rifampin. All antimicrobials showed strong bactericidal activity against *in vitro*-grown cultures in CFU assays, with counts below the limit of detection (>6 log_10_ reduction) at day 7 for isoniazid and streptomycin for H37Rv (Fig. 5A and B, black bars). After passage, our sputum isolates showed markedly increased susceptibility to killing (Fig. 5A and B, white and gray bars). Treatment of samples S6 and S20 cultured isolates with streptomycin, isoniazid, ethambutol, or rifampin for 7 days resulted in dramatic reductions of CFU counts that were at least an order of magnitude greater than seen with the direct tests applied to the sputum samples.

Genotyping by MIRU-VNTR (15) revealed the following lineages: East African Indian (S6), Haarlem (S19), previously unknown (S20), and Beijing (S21).

### Inoculation of a passaged M. tuberculosis isolate into sputum followed by decontamination and freezing does not result in tolerance.

**FIG 6:**
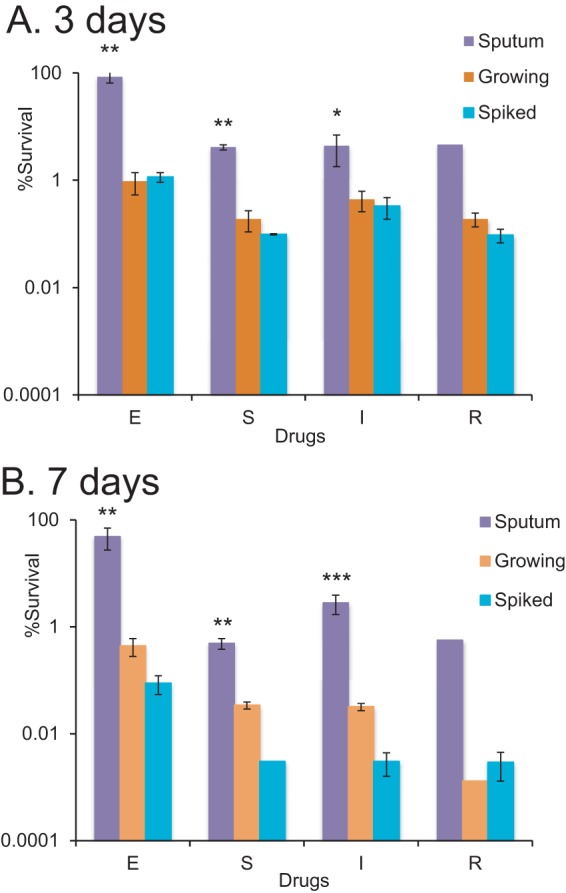
Plateable M. tuberculosis in sputum is more tolerant to drug treatment than corresponding isolates processed *in vitro*. The M. tuberculosis isolate from sputum sample S20 was grown in 7H9 medium to logarithmic phase. These mycobacteria were decontaminated and frozen in glycerol (orange bars); the same bacteria were spiked into nontuberculosis sputum, decontaminated, and frozen in glycerol (turquoise bars). After defrosting, mycobacteria were treated with drugs for 3 (A) and 7 (B) days before assessment of CFU counts. These counts were compared to those obtained in the direct sputum tolerance assay for sputum sample S20 (lilac bars). S, streptomycin, 20 μg/ml; E, ethambutol, 20 μg/ml; I, isoniazid, 10 μg/ml; R, rifampin, 5 μg/ml. **, survival values for M. tuberculosis treated directly in sputum were statistically significantly different from those of the treated sputum isolate or spiked sputum isolate (*P* < 0.01, one-way ANOVA).

To determine whether sputum processing and storage contributed to the drug tolerance observed, we decontaminated and froze *in vitro*-grown S20 cultures directly and after inoculation into an M. tuberculosis-negative sputum. The antimicrobial tolerance of the M. tuberculosis isolate in these preparations was compared with that found in the direct sputum analysis by CFU assay (Fig. 6). Decontamination and freezing increased the tolerance of *in vitro*-grown M. tuberculosis; however, the proportions that survived in the direct sputum analysis were still significantly higher (1.5 to 2 log_10_).

It was not possible assess the effects of processing on tolerance in the nonplateable and SN-dependent populations, as these populations are lost on passage (6).

## DISCUSSION

M. tuberculosis is notoriously difficult to treat and eradicate from infected patients. It adopts multiple physiological states and can survive *in vivo* and *in vitro* for decades (20). While prolonged treatment of TB patients with a combination of four first-line drugs is effective, the duration of therapy is a minimum of 6 months. Recent drug trials designed to reduce the duration of treatment have had limited success (21, 22). These challenges emphasize the importance of developing preclinical tests for candidate drugs, including *in vitro* models that better predict clinical efficacy. *In vitro* systems for the assessment of drug effects on growing and nongrowing M. tuberculosis have recently been reviewed, but the artificial nature of these assays and high variability of results limit their utility for predicting clinical treatment outcomes (23).

We have described a simple system which can be used for testing drugs directly using *ex vivo* M. tuberculosis cells obtained from patients with TB. We demonstrated that the quantitative balance between mycobacterial populations in sputum can be preserved during storage at −80°C, enabling further standardization of protocols and replicate analyses. Use of freeze-dried batches of culture supernatants minimizes variations in resuscitation activity. Rpf-containing culture supernatant could be useful for formulation of media for assessment of M. tuberculosis from treated patients, as Rpf-dependent bacilli have been isolated from samples that were negative by conventional culture (6, 24, 25). Determining the duration of treatment for tuberculosis is problematic and is not aided by molecular tests, such as the XpertMTB/RIF test (26). However, M. tuberculosis culture supernatant has been shown to accelerate growth of mycobacteria from treated sputum samples (27). Assessment of sputum samples from patients during tuberculosis treatment using Rpf-supplemented medium may provide information relevant to determining endpoints for therapy with minimal risk of relapse. The standardization of protocols and the storage procedure described here will enable independent evaluation of sputum samples at multiple sites.

Our findings further support previously published results on the phenotypic adaptation of mycobacteria during infection and the transition in sputum (4, 6). The numerically dominant SN-dependent M. tuberculosis populations in sputum are more tolerant to isoniazid and streptomycin. Pyrazinamide had no substantial activity in killing SN-dependent mycobacteria in sputum, while ethambutol showed only marginal killing activity against this population. Longer exposure to these drugs may be required for detection of substantial killing activity. Alternatively, the conditions used in our assay did not replicate the *in vivo* conditions that are necessary for killing of M. tuberculosis by ethambutol and pyrazinamide. Despite the variable response to rifampin, our data suggest the presence of populations of SN-dependent M. tuberculosis cells tolerant to this agent in sputum. One reason for the variability observed here may have been the use of relatively low drug concentrations in the assay mixtures. Both degradation of rifampin (reported half-life of 9 days at 37°C in 7H10 agar [28]) and subtle differences in susceptibility of different M. tuberculosis strains in sputum may have contributed to our results. Possible alterations in the composition of sputum proteins and their binding to rifampin may also have influenced the killing of M. tuberculosis.

We note with interest that plateable M. tuberculosis bacilli in sputum were also more tolerant to all drugs tested than was H37Rv; this property was lost upon passage *in vitro*. While we had previously thought that tolerance was particularly associated with the SN-dependent population (6), our results indicated that the plateable population also shows properties *ex vivo* that differ from *in vitro*-grown M. tuberculosis. Drug-tolerant M. tuberculosis and Mycobacterium marinum were previously observed during macrophage infection, and this property was attributed to the activation of efflux pumps (29). We have recently shown that the *in vivo* environment accelerates generation of Rpf-dependent mycobacteria (7). SN-dependent M. tuberculosis isolates described in the present study are likely synonymous with the Rpf-dependent M. tuberculosis previously detected in sputum (6); however, we cannot exclude that other factors in culture supernatant may contribute to recovery of M. tuberculosis in culture. These potentially overlapping populations that are dependent on factors present in culture supernatant may represent nongrowing, metabolically active forms produced during stress and exposure to host immunity (8, 30). Their biological significance awaits clarification; nonetheless, the relatively high numbers of SN- and Rpf-dependent M. tuberculosis cells detected in sputum and their drug tolerance indicate their likely important contribution to the necessity for prolonged treatment. This proposal has received recent support from the striking observation of Hu and colleagues that elimination of Rpf-dependent M. tuberculosis by treatment with high-dose rifampin prevented TB relapse in Cornell model mice (31).

The potential significance of the M. tuberculosis populations studied here for the analysis of outcome of treatment raises the question of their use in assessing clinical management. The recent demonstrations of the potential significance of lipid body assessments in predicting the outcome of therapy (32) increases the incentive to develop analytically more amenable biomarkers of tolerant M. tuberculosis populations, such as the molecular bacterial load assay (33) or transcriptomic profiling (34).

**FIG 7:**
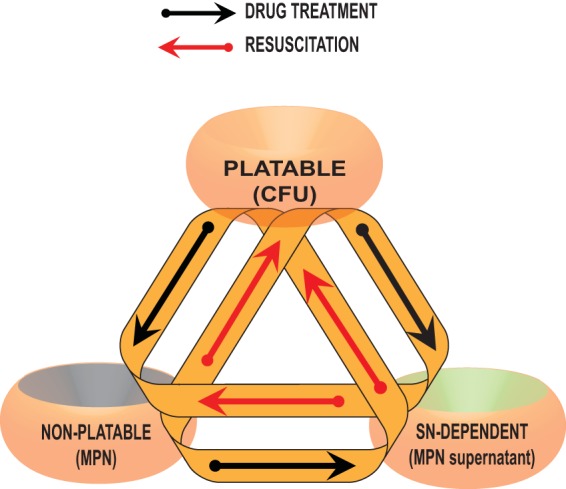
Suggested transitions between M. tuberculosis populations *in vivo* and during the sputum drug tolerance assay. Note that there may be substantial overlap between the populations such that all the colony-forming cells are propagated in the MPN and MPN_SN assays.

Interpretation of our results is limited by the restricted range of drug concentrations used and by likely transitions of cells between the M. tuberculosis populations detected by our three assays during the test. As shown in Fig. 7, plateable, nonplateable, and SN-dependent populations are highly dynamic and are influenced by drug treatment (black arrows) and potential resuscitation during incubation in liquid medium (red arrows). Our preliminary data suggest that drug treatment may induce Rpf dependency, while Rpf-dependent M. tuberculosis may convert into plateable forms during incubation in liquid. We hypothesize that these processes happen *in vivo* and determine the outcome of infection and treatment.

The findings of this successful proof-of concept study offer a novel opportunity for evaluation of drug candidates using *ex vivo* M. tuberculosis cells and for assessment of individuals' responses to treatment. Our simple system enables investigation of differential responses attributable to distinct M. tuberculosis populations generated during the course of infection and treatment in the natural host. Thus, we suggest the system has significant potential to improve predictions of clinical outcome and to enable shortened treatment periods.

## Supplementary Material

Supplemental material
