# Supplementary material for: Phenotypically Adapted Mycobacterium tuberculosis Populations from Sputum Are Tolerant to First-Line Drugs
Source: Antimicrob Agents Chemother. 2016 Mar 25;60(4):2476–83. doi: 10.1128/AAC.01380-15 (PMC4808147; doi:10.1128/AAC.01380-15)
Supplement: Supplemental material [file supp_60_4_2476__index.html]

Phenotypically Adapted Mycobacterium tuberculosis Populations from Sputum Are Tolerant to First-Line Drugs — Supplemental material 

# Phenotypically Adapted Mycobacterium tuberculosis Populations from Sputum Are Tolerant to First-Line Drugs

## Supplemental material

- Supplemental file 1 -

  Supplemental Table S1 and Figures S1 and S2

  PDF, 587K
