# Supplementary material for: Phenotypically Adapted Mycobacterium tuberculosis Populations from Sputum Are Tolerant to First-Line Drugs
Source: Antimicrob Agents Chemother. 2016 Mar 25;60(4):2476–83. doi: 10.1128/AAC.01380-15 (PMC4808147; doi:10.1128/AAC.01380-15)
Supplement: Supplemental material [file AAC.01380-15_zac004165082so1.pdf]

Phenotypically adapted *Mycobacterium tuberculosis* populations from sputum are tolerant to first line drugs

Obolbek Turapov[1], Benjamin O'Connor[1], Asel A. Sarybaeva[1], Caroline Williams[1], Hemu Patel[2], Abdulat Kadyrov[3], Akpay S. Sarybaev[4], Gerrit Woltmann[5], Michael R. Barer[1, 2], Galina V. Mukamolova[1*].

[1]Department of Infection, Immunity and Inflammation, University of Leicester, Leicester, LE1 9HN, UK; [2]Empath Pathology Services, Department of Clinical Microbiology, University Hospitals of Leicester NHS Trust, Leicester, UK

[3]National Centre of Phthisiology, Akhunbaeva Str 90A, Bishkek, Kyrgyzstan,

[4]Kyrgyz Indian Mountain Medical Research Center, Bishkek, Kyrgyzstan

[5]Department of Respiratory Medicine, Glenfield Hospital, Leicester, UK

*To whom correspondence should be addressed. E-mail: gvm4@le.ac.uk

Content

Table S1. Influence of storage medium on Mtb viability in frozen sputum samples.

Figure S1. Freeze-dried culture supernatants retain resuscitation-promoting activity.

Figure S2. Reproducibility of growth assay using sputum samples and H37 Rv.

Sputum samples	Log10 cells/ml			
	Fresh	Frozen in PBS	Frozen in trehalose	Frozen in glycerol
S1 CFU	1.3	1.95	1.69	1.69
S1 MPN	1.0	1.66	1.66	1.66
S1 MPN _SN	3.8	4.34	4.49	4.62
S2 CFU	1.0	1.0	1.0	1.0
S2 MPN	1.3	2.66	2.66	2.66
S2 MPN _SN	3.8	3.34	3.51	4.41
S3 CFU	3.04	3.47	3.32	3.61
S3MPN	2.66	2.66	2.66	2.66
S3 MPN _SN	4.73	4.73	4.83	4.91
S4 CFU	4.09	4.06	4.06	4.25
S4 MPN	1.66	1.66	1.66	1.66
S4 MPN _SN	4.85	4.34	4.66	5.15

Table S1. Influence of storage medium on Mtb viability in frozen sputum samples.

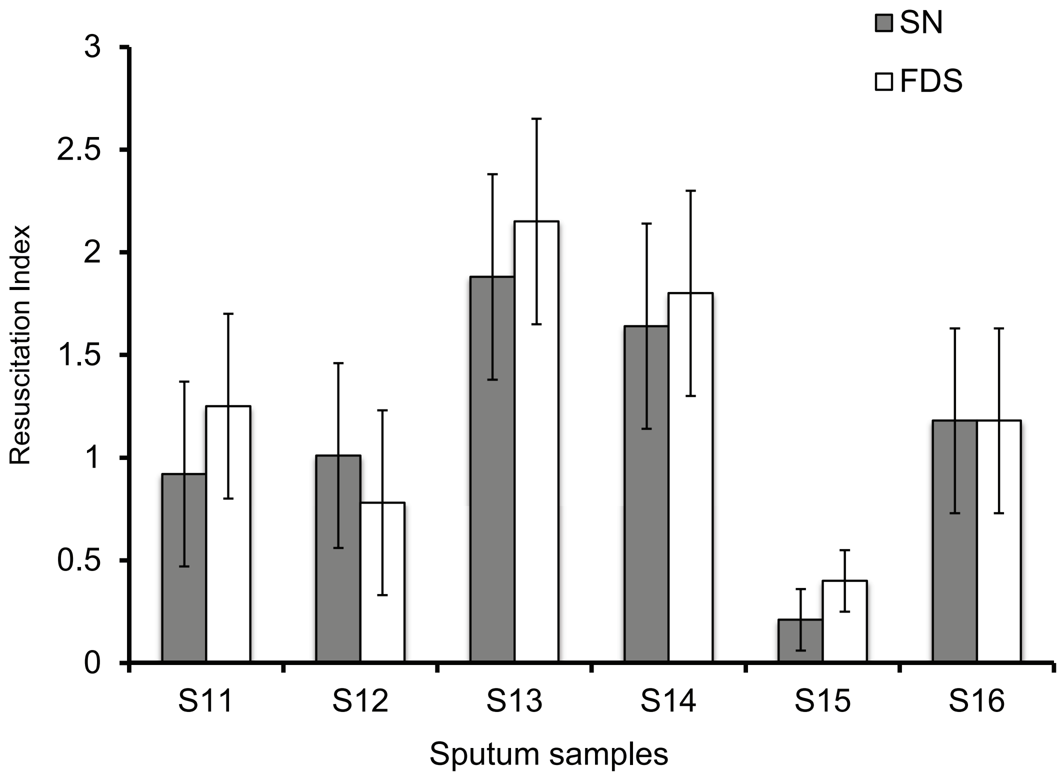

Figure S1. **Freeze-dried culture supernatants retain resuscitation-promoting activity.** Culture supernatant was prepared as described in material and methods. Resuscitation activities of freshly prepared (SN) and freeze-dried (FDS) supernatants were assessed and expressed as resuscitation index RI = (logMPN_SN – logCFU). Error bars indicate 95% limits of confidence for MPN counts. No statistically significant difference was observed between resuscitation activities of freshly prepared and freeze-dried culture supernatants ($p>0.05$, t test).

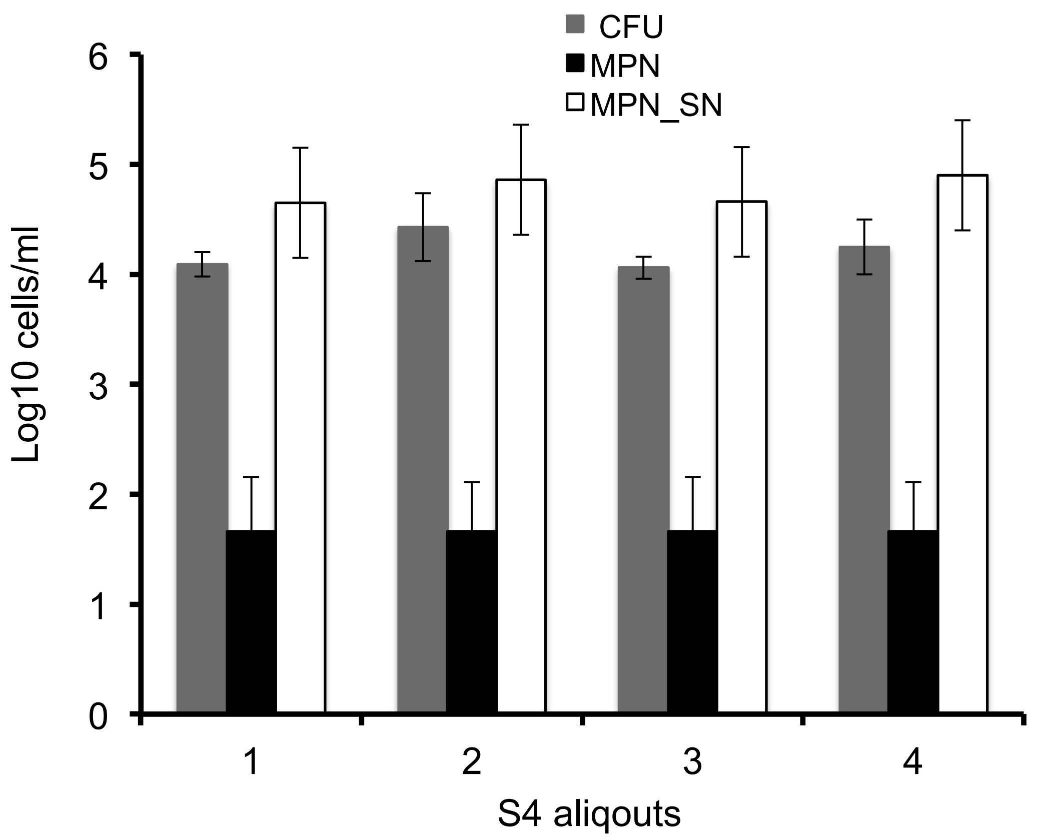
CFU
MPN
MPN_SN
Log10 cells/ml
0
1
2
3
4
5
6
1
2
3
4
S4 aliqouts

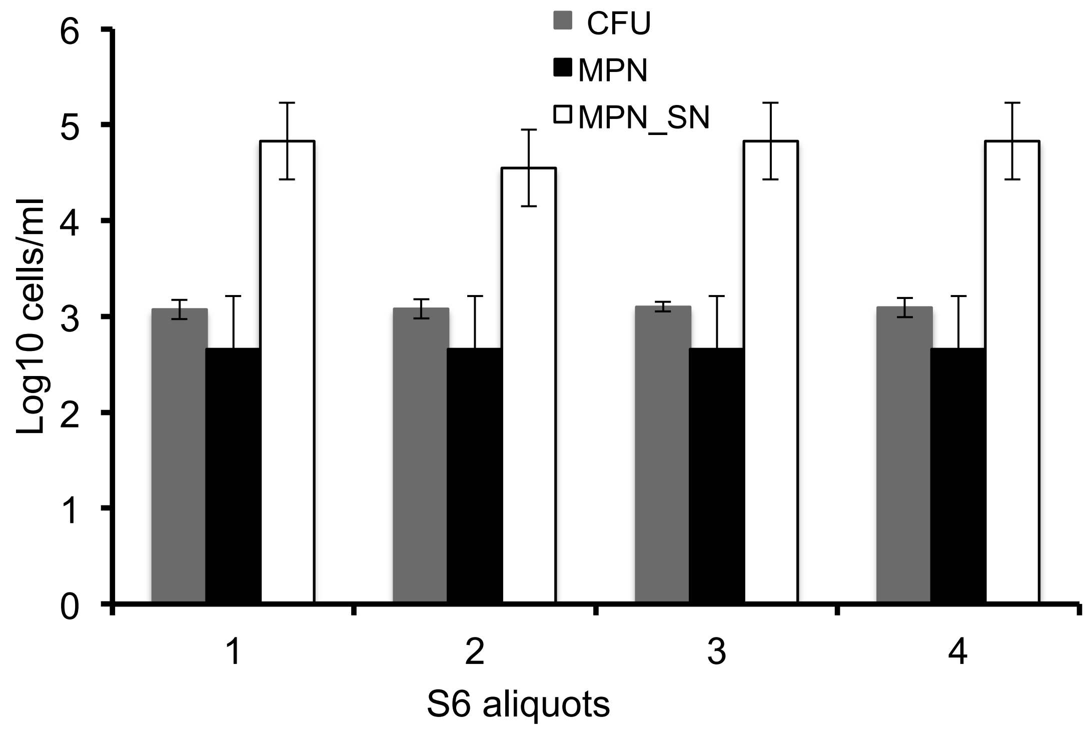
CFU
MPN
MPN_SN
Log10 cells/ml
0
1
2
3
4
5
6
1
2
3
4
S6 aliquots

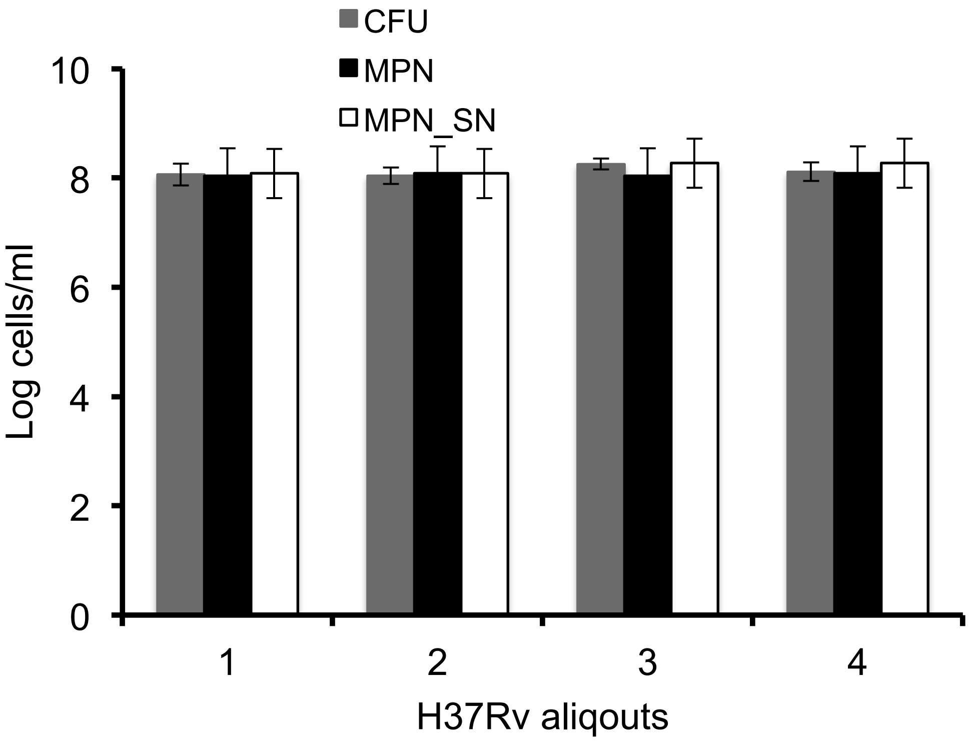

Figure S2. Reproducibility of growth assay using sputum samples and H37 Rv. Mycobacteria were decontaminated, resuspended in 10% glycerol and stored at -80°C. Four separate aliquots were defrosted and assessed separately. A. *Mtb* from sputum sample S4. B. Mtb from sputum sample S6. C. H37Rv laboratory strain.
